# The association of heavy and light chain variable domains in antibodies: implications for antigen specificity

**DOI:** 10.1111/j.1742-4658.2011.08207.x

**Published:** 2011-08

**Authors:** Anna Chailyan, Paolo Marcatili, Anna Tramontano

**Affiliations:** 1 Department of Physics, Sapienza University of RomeItaly; 2 Istituto Pasteur Fondazione Cenci Bolognetti, Sapienza University of RomeItaly

**Keywords:** antigen binding, immunoglobulins, interface, structure analysis, variable domain packing

## Abstract

The antigen-binding site of immunoglobulins is formed by six regions, three from the light and three from the heavy chain variable domains, which, on association of the two chains, form the conventional antigen-binding site of the antibody. The mode of interaction between the heavy and light chain variable domains affects the relative position of the antigen-binding loops and therefore has an effect on the overall conformation of the binding site. In this article, we analyze the structure of the interface between the heavy and light chain variable domains and show that there are essentially two different modes for their interaction that can be identified by the presence of key amino acids in specific positions of the antibody sequences. We also show that the different packing modes are related to the type of recognized antigen.

## Introduction

Immunoglobulins are multi-chain proteins usually consisting of two pairs of light chains and two pairs of heavy chains (with the remarkable exception of ‘heavy chain antibodies’, which are found in camelids [1] and in a number of fishes [2,3], and are devoid of light chains).

In higher vertebrates, there are two types of light chain –κ and λ– whereas heavy chains can be of five types: μ, δ, γ, ε and α. The type of heavy chain defines the class of immunoglobulin: IgM, IgD, IgG, IgE and IgA, respectively. Each chain contains four (heavy chains) or two (light chains) intrachain disulfide bonds and is composed of multiple variants of a basic domain (two for the light and usually four for the heavy chain) assuming the characteristic immunoglobulin fold, in which two β-sheets are packed face to face and linked together by conserved interchain disulfide bridges and by interstrand loops.

On the basis of the sequence analysis of several antibodies, Wu and Kabat [4] correctly predicted that six loop regions (three from the light and three from the heavy variable domains) are involved in antigen binding, and called them ‘complementarity determining regions’ or CDRs. This sequence-based definition largely overlaps with the structural definition of the ‘hypervariable loops’ subsequently provided by Chothia *et al.* [5].

The regions of the variable domains outside these loops are called the framework, and are highly conserved in both sequence and main-chain conformation, whereas the six loops of the antigen-binding site, primarily responsible for recognizing and binding the antigen, are more variable in sequence and structure. Antibody fragments obtained by limited proteolytic digestion, which contain only a subset of the domains of a complete antibody, maintain either the antigen-binding ability [antigen-binding fragment (Fab), two connected Fabs (F(ab)2), variable fragment (Fv)] or the effector functions (Fc, hinge) [6].

There is great interest in correctly predicting the structure and specificity of these molecules, given their essential role in the physiological immune response, as well as in relevant disease processes. Furthermore, their modular nature and the conservation of their scaffold structure make antibody molecules particularly suitable candidates for protein engineering. It is possible to ‘transplant’ the antigen-binding property from a ‘donor’ to an ‘acceptor’ antibody by exchanging either fragments or antigen-binding regions. In this way, the specificity of an antibody against a given antigen, obtained for example in the mouse, can, in principle, be transferred to a human antibody, thereby obtaining a molecule with the desired specificity and less likely to elicit an immune response. Several strategies have been devised to reach this goal, such as antibody chimerization [7], humanization [8,9], superhumanization [10,11], resurfacing [12] and human string content optimization [13]. All of these methods rely on a correct understanding of the relationship between sequence and structure in this class of molecule.

We and others have contributed to the development of the canonical structure method to predict the structure of the hypervariable loops [5,14–16]. This method is based on the observation that, in spite of their high sequence variability, five of the six loops of the antigen-binding site, and part of the sixth, can assume a small repertoire of main-chain conformations, called ‘canonical structures’, determined by the length of the loops and by the presence of key residues at specific positions, inside and outside of the loops themselves. The other loop residues are free to vary to modify the topography and physicochemical properties of the antigen-binding site. Most of the hypervariable regions of known structures have conformations very close to the described canonical structures [5,14]. The method is implemented in the publicly available web server PIGS [17] and has been extended recently to allow the prediction of the structure of loops from immunoglobulin λ chains [15].

Previous studies [18–21] have shown that changes in the heavy chain variable domain–light chain variable domain (VH–VL) association can modify the relative positions of the hypervariable loops, which, in turn, can alter the general shape of the antigen-binding site, as well as the disposition of side-chains that interact directly with the antigen [22–25].

In 1985, Chothia *et al.* [26] proposed a model for the association of VH and VL, taking into account the interface geometry and the packing of residues involved in the interaction. However, the study was based on only three crystallographic structures. More recently, attempts to study and predict the VH–VL packing geometry [27–29] have led to the conclusion that a large number of residues from both the framework and the hypervariable loops contribute to the tuning of the interface geometry.

In this article, we present a comprehensive analysis of the VH–VL interface of several experimental structures of immunoglobulins currently available. We show that there are two fundamentally different modes of interaction between the domains. Notably, we also identify the specific sequence features associated with the two geometries and highlight the effect of the different packing modes on the size of the recognized antigen.

## Results

**Fig 1:**
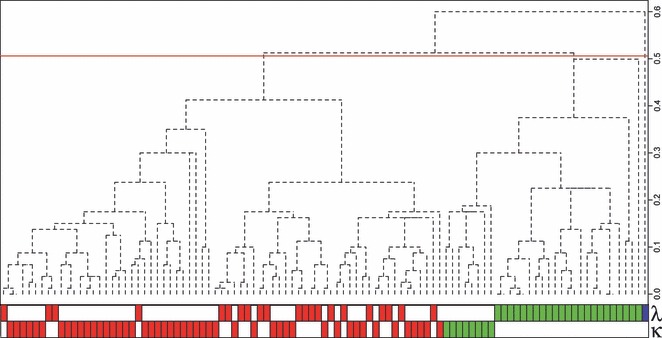
Results of the cluster analysis. Dendrogram based on the difference between the positions of residues at the interface in the light and heavy chain variable domains. The red line indicates the clustering with the highest silhouette value (0.47). In the bottom panel, red, green and blue indicate the A, B and C clusters, respectively. The type of light chain is shown in the bottom panel.

A nonredundant dataset of immunoglobulins of known structure taken from the Protein Data Bank (PDB) [30], balanced in terms of light chain type, was constructed, as described in the Materials and methods section, and contains 101 immunoglobulin structures (56 antibodies with κ- and 45 antibodies with λ-type light chains). We applied several clustering methods to the immunoglobulins of this dataset, all based on the structural distance among the residues contributing to the interface. The diana divisive clustering method (M. Maechler, P. Rousseeuw, A. Struyf and M. Hubert, unpublished results) was selected as the best performing technique on the basis of the corresponding silhouette value [31] (see Materials and methods section for details), and produced three clusters (Fig. 1).

The first cluster (hereafter referred to as cluster A) contains 69 immunoglobulin structures, the second (cluster B) contains 31 immunoglobulin structures and the third (cluster C) is formed by a single antibody structure (PDB code: 1Q1J).

The interface of 1Q1J does not resemble any other structure in our dataset. Its residues have a root-mean-square deviation (RMSD) of about 1.4 Å from the residues contributing to the interface of a cluster A representative structure (PDB code: 2ORB) and about 1.4 Å from those of a cluster B representative structure (PDB code: 2A6I).

1Q1J is the structure of the human monoclonal antibody 447-52D complexed with a peptide derived from the V3 region of the HIV-1 gp120 protein. Another structure (PDB code: 3C2A) for the same antibody, bound to a variant of the same peptide, is available and has an interface essentially identical to that of 1Q1J. This is the only antibody in our set that uses the heavy chain V gene IGHV3-15. Its uniqueness did not allow us to analyze it further.

There is no strong correlation between the structural clustering and the type of light chain. λ and κ chains contribute to both clusters, and therefore the structural difference in the interface cannot be attributed to the type of light chain (Fig. 1).

**Fig 2:**
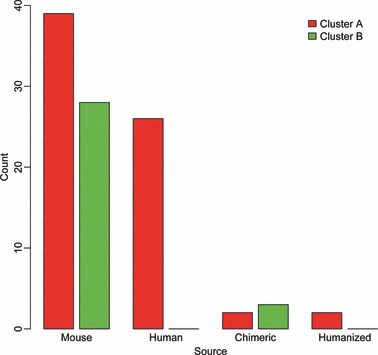
Antibody source. Frequency of mouse, human, chimeric and humanized antibodies in clusters A (red bars) and B (green bars).

Cluster A is formed by immunoglobulins from both mouse and human, whereas cluster B is only populated by immunoglobulins from *Mus musculus* (28 immunoglobulins) and by chimeric antibodies with a mouse variable domain and a human constant domain (three immunoglobulins) (Fig. 2). This implies, as discussed later, that some packing modes observed in mouse antibodies cannot be found in human antibodies, with obvious implications for humanization experiments.

We observed a bias in the usage of light chain V germline genes, whereas this was not the case for the heavy chain V genes. There is no intersection between the light chain germlines used in cluster A and those used in cluster B. The latter set of germlines is enriched in λ-type light chains [IGLV1 (23/31)], even though a number of κ-type light chains [IGKV10-94 (2/31), IGKV10-96 (4/31), IGKV9-124 (1/31), IGKV14-100 (1/31)] are found in the cluster. In cluster A, the numbers of immunoglobulins of λ and κ type are 21 and 48, respectively. In other words, there is a mode of interaction between the two chains characteristic of the immunoglobulins of cluster B, specific for a subset of mouse immunoglobulins and never observed in humans (Table S1).

Our next step involved the investigation of whether the structural difference in the packing of the two domains could be ascribed to the presence of specific amino acids. To this end, we used the Random Forest technique [32] (see also Materials and methods section) to evaluate the relative ability of each residue to identify the structural cluster to which the immunoglobulin belongs. The Gini index [33], a measure of the importance of the sequence positions, was used to select the most significant. The eight sequence positions with the largest Gini index, described and analyzed in detail below, are able to discriminate between the two clusters with a classification error lower than 10%. These positions (listed here in order of their relevance) are L44, L43, L41, L42, L8, L28, L66 and L36.

**Fig 3:**
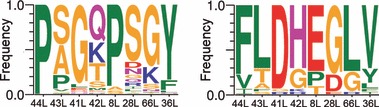
Logo of discriminative positions. Sequence logos [34] for the positions highlighted as discriminative for clusters A (left side) and B (right side) by the Gini index analysis in the structure dataset. The height of the letters is proportional to the frequency of the corresponding amino acid in the position indicated on the *x* axis. The letters are colored according to the scheme used in Lesk [35]. Orange: small nonpolar G, A, S, T; green: hydrophobic C, V, I, L, P, F, Y, M, W; magenta: polar N, Q, H; red: negatively charged D, E; blue: positively charged K, R.

The sequence logo for all eight positions [34] (Fig. 3) clearly shows that immunoglobulins belonging to different clusters have different preferences for specific amino acids in these positions. It should be mentioned that cluster B is formed by a large fraction (23 of 31) of mouse immunoglobulins with a λ chain from the IGLV1 germline, and three of the positions highlighted by the Random Forest analysis (L8, L28 and L66) are completely conserved in all sequences of this type. Furthermore, none of them is in contact with the heavy chain. This strongly suggests that they discriminate this particular type of λ chain from all the others and are not specific for the type of interface.

The remaining five positions (L41–L44 and L36) are instead located at the interface between the two chains, and the difference in the amino acids occupying them is likely to be related to the packing of the domains.

In particular, position L44 is always occupied by a proline in immunoglobulins belonging to cluster A, whereas a medium/large hydrophobic amino acid is preferred in the equivalent position in cluster B (Table 1). Proline L44 in cluster A adopts a *trans* conformation and interrupts the β-strand regularity preserved in cluster B. This affects the type of turn observed in the two clusters: the region L41–L43 forms a tight turn (typically a 3 : 3 class hairpin conformation) connecting the two proximal β-strands in immunoglobulins belonging to cluster B. Conversely, a 7 : 7-type hairpin is present between residue L38 and residue L44 in cluster A.

**Table 1:** Amino acid occurrence at positions L36, H100X and L44 in immunoglobulins belonging to clusters A and B.

	Cluster A	Cluster B
Position	Amino acid: occurrences	Amino acid: occurrences
L36	Y: 58 F: 8 L: 2 N: 1	V: 22 Y: 5 L: 2 F: 1 I: 1
H100X	F: 28 M: 21 V: 5 S: 4 P: 4 G: 3 L: 3 I: 1	F: 14 M: 7 G: 5 L: 4 S: 1
L44	P: 69	F: 24 V: 5 I: 2

In all immunoglobulins, residue L44 interacts with the amino acid at position L36, which is a large amino acid in most of the members of cluster A, and usually smaller, typically a valine, in those belonging to cluster B (Table 1).

The side-chain of residue L36 packs against the last insertion before residue H101 (which has a different numbering according to the specific structure and is called H100X here for clarity), which is, in most cases, a phenylalanine or a methionine. A different frequency of residues in position H100X is observed in clusters A and B (Table 1).

The packing between residues L36 and H100X is different in the two clusters. We computed the distribution of the distances between the residue 36 Cα of the light chain and that of residue 100X of the heavy chain. In cluster A, the average is 9.79 Å with a standard deviation of 1.36 Å, whereas the corresponding values for cluster B are 8.22 and 1.17 Å, respectively. The two distributions are statistically significantly different (*P* = 1.3 × 10^−6^).

**Fig 4:**
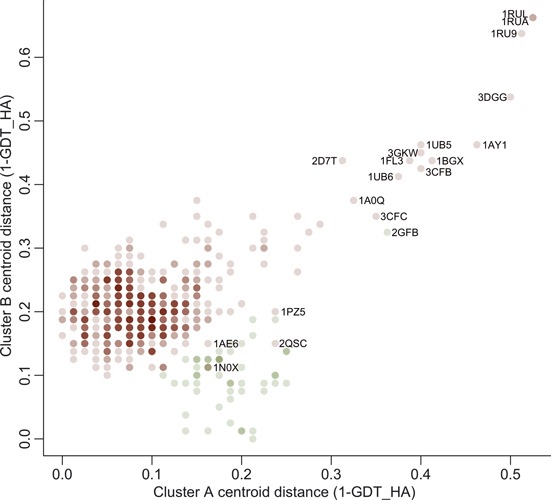
Interface distance plot of antibodies not included in the original dataset. Plot of the distance (1 – GDT_HA) between the Cα of the 20 residues at the VH–VL interface of the immunoglobulins not originally included in the nonredundant structure dataset and the corresponding atoms of the centroids of clusters A and B. Red dots indicate immunoglobulins in which position L44 is occupied by a proline. Outliers are labeled and discussed in the text.

The presence of a proline in position L44 is the best predictor of the presence of a type A interface. We computed the distance between the Cα of the residues contributing to the interface and the corresponding residues of the centroid of clusters A (PDB code: 2ORB) and B (PDB code: 2A6I) for all the immunoglobulins of known structure that were left in our initial nonredundant dataset (584 antibodies), and plotted one against the other (Fig. 4). Almost all of the immunoglobulins that contain a proline in position L44 are more similar to those of cluster A (515/533). A few immunoglobulins have an interface that is different from those observed in both clusters. Fourteen are expected to adopt a type A interface because they have a proline at position L44 (PDB codes: 1BGX, 1AY1, 1FL3, 3CFC, 3CFB, 1UB5, 1UB6, 1RUL, 1RU9, 1RUA, 3DGG, 1A0Q, 2D7T and 3GKW) but do not, and only one (PDB code: 2GFB) does not have the expected type B interface, although the proline in position L44 is not present. In the first seven cases, the structures are either not well resolved or have a high *B* factor. 1RUL, 1RU9 and 1RUA are solved structures of the same antibody after UV irradiation. The same nonirradiated antibodies (PDB codes: 1NCW and 1ND0) display the normal interface and are properly classified in cluster A. In 3DGG, a magnesium ion coordinates several residues in the region L39–L46 distorting the loop. 1A0Q is a catalytic antibody with esterase activity that contains a ligand (*S*-norleucine phenyl phosphonate) deeply buried in the binding site. The last three cases (PDB codes: 2D7T, 3GKW and 2GFB) seem to be genuine outliers.

Two more structures of antibodies containing a proline in position L44, (corresponding to entries 1PZ5 and 1N0X) are more similar to cluster B. However, there are different determinations of their structures with different ligands and in these cases the interface packing follows the rules outlined here. In 1AE6, the proline is present, but in a *cis* conformation, and the region has a very high *B* factor. A high *B* factor is also observed for the whole 2QSC molecule.

**Fig 5:**
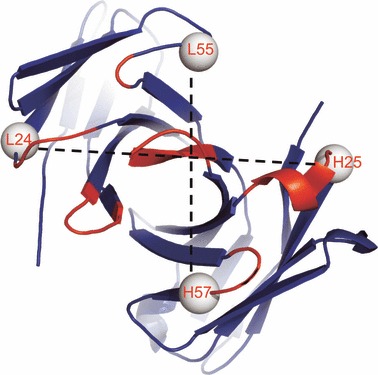
Antigen-binding site dimensions. Positions of the residues used to estimate the width of the antigen-binding site in the two clusters. The Cα moieties of the selected residues (L55, H57, L24 and H25) are indicated by spheres. Broken lines indicate the measured distances. The structure shown is the PDB entry 2FL5.

The next question we asked is whether the difference in the packing geometry observed in the two clusters has an impact on the conformation of the antigen-binding site. We selected two pairs of residues on opposite sides of the binding site (L55 and H57; L24 and H25, Fig. 5) and computed the distribution of the distances between their Cα atoms in immunoglobulins belonging to clusters A and B.

The average distance between L55 and H57 is 26.49 ± 0.98 Å in cluster A and 24.82 ± 1.39 Å in cluster B. The corresponding values for L24 and H25 are 35.87 ± 0.65 Å and 34.95 ± 0.58 Å for clusters A and B, respectively, corresponding to a difference of about 10% in the area of the rhomboid defined by the four Cα atoms. The two distributions are statistically significantly different (*P* = 1.9 × 10^−7^ and *P* = 2.9 × 10^−3^ for the first and second pair, respectively). In some cases, the antibodies included in our dataset were solved in a complex with their antigen (71 of 101 cases). To exclude the possibility that the presence of the antigen is responsible for the observed differences in the distance distributions, we recalculated them by considering bound and unbound antibodies separately (Table 2). The observed differences are still present and still statistically significant. This implies that, on average, the binding site of the type A immunoglobulins is wider than that of the type B immunoglobulins.

**Table 2:** Average distances between residues L55–H57 and between residues L24–H25 in all immunoglobulins belonging to clusters A and B. The table also shows the values for bound (holo-form) and unbound (apo-form) cases separately.

		L55–H57 distance (Å)	L24–H25 distance (Å)
Total dataset (100)	Cluster A (69)	26.49 ± 0.98	35.87 ± 0.65
Cluster B (31)	24.82 ± 1.39	34.95 ± 0.58
Holo-form (70)	Cluster A (45)	26.51 ± 0.94	35.87 ± 0.57
Cluster B (25)	24.62 ± 1.34	34.96 ± 0.63
Apo-form (30)	Cluster A (24)	26.45 ± 1.08	35.89 ± 0.8
Cluster B (6)	25.62 ± 1.45	34.95 ± 0.34

In 71 cases in our dataset, the structure of the immunoglobulin has been determined in a complex with an antigen. We computed the volume of these antigens and classified them into two groups as described in the Materials and methods section. Clusters A and B contain 46 and 25 immunoglobulins complexed with an antigen, respectively. Among the 17 that are bound to a small antigen (volume < 505 Å^3^), 14 belong to cluster B and only three to cluster A. Such a difference is statistically meaningful (*P* = 6.9 × 10^−6^; see Materials and methods section for details). It is therefore evident that antibodies belonging to cluster B generally bind smaller antigens, whereas those in cluster A are more promiscuous. For comparison, the *p*-nitrophenyl-phosphocholine molecule (molecular formula: C_11_H_18_N_2_O_6_P; PDB code: 1DL7) is a simple hapten and has a volume of 451 Å^3^, whereas the nine-residue rhodopsin epitope mimetic peptide (sequence TGALQERSK; PDB code: 1XGY) has a volume of 809 Å^3^. In practice, this threshold discriminates small hapten-like antigens from peptide and protein antigens.

In summary, the results of the analysis described here clearly indicate that there are at least two different packing modes for the association between the light and heavy domains in immunoglobulins, and these can be specifically associated with key residues in their sequence.

**Fig 6:**
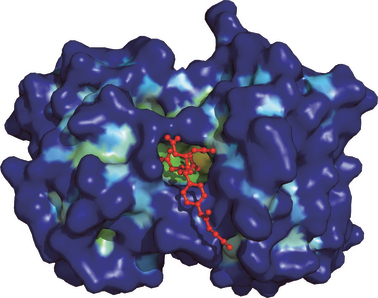
Antigen-binding site of type B antibody. Molecular surface of the antigen-binding site of the CHA255 antibody (PDB code: 1IND). The presence of a rather narrow pocket is clearly visible. The surface is colored according to the atom depth (using the DPX web server [36]); the ligand (indium chelate) is depicted in red using a ball and stick representation.

Importantly, the two different packing modes have a significant effect on the geometry of the binding site, as illustrated by the statistically significantly different distribution of distances between residues at the periphery of the binding site, and we have shown that these differences are related to the size of the recognized antigen. Furthermore, visual analysis indicates the presence of a narrow pocket in the middle of the binding site in the majority of the immunoglobulins of cluster B (Fig. 6).

## Discussion

The results presented here are clearly relevant for antibody and antibody library design, but also for humanization experiments. The type B interface is only found in the mouse, and therefore grafting the antigen-binding site of a type B murine antibody into a human antibody will be ineffective if the recipient molecule has a type A interface. One instructive example can be found in the work by Worn *et al.* [37]. These authors produced two single-chain Fv humanized intrabody versions of a murine anti-GCN4 immunoglobulin molecule (with a λ chain) using, as recipient, two human antibodies that differed in the type of light chain (λ in one case and κ in the other) and in only seven residues (including residues L36, L43 and L44). The λ-graft variant had an activity comparable with the wild-type antibody, whereas the κ-graft variant, although extraordinarily stable *in vitro*, had a five order of magnitude decreased antigen affinity, presumably, as the authors suggest, caused by differences in the mutual orientation of the two domains.

Finally, we would like to mention that the ability of type B antibodies to bind smaller antigens, and the presence of the pocket described, might open up the possibility of using them as potential drug delivery vectors. Indeed, this has been proposed already in the case of the 1IND antibody [38], a type B immunoglobulin with an exceptionally high affinity binding for an indium-chelate hapten.

The ability to use sequence data to predict the mode of association of the variable domains of antibodies also has implications for methods to predict their structure. Indeed, the information obtained through the analysis described here is being used to implement a better prediction protocol in our immunoglobulin structure prediction server [17].

## Materials and methods

Throughout this article, we have used the Kabat–Chothia numbering scheme [39] with the additional insertion at position L68 proposed by Abhinandan and Martin [40]. The letters L and H preceding a residue number indicate light and heavy chain residues, respectively.

We constructed a dataset of immunoglobulins of known structure containing both λ and κ chains. Starting from 120 structures with λ-type light chains, downloaded from the PDB database [30], version 21st February 2010, we removed single-chain immunoglobulins (34), single-chain variable fragments (5), redundant structures (i.e. structures for which both the light and heavy chain variable regions, if present, are identical in sequence) (26) and the ten structures with resolution worse (higher) than 3 Å (using the PISCES web server [41]). The final set contained 45 immunoglobulins of known structure with a λ light chain. The number of known structures of immunoglobulins with a κ-type light chain stored in PDB is much higher (930). We removed all single-chain immunoglobulins and light chain dimers, and subsequently only retained those with a resolution better than 3 Å (using the PISCES web server [41]). This resulted in a set of 640 structures with κ light chains. In order to obtain a balanced dataset for κ and λ light chains, whilst, at the same time, preserving diversity among the κ light chains, we grouped together immunoglobulins with κ light chains with similar residues in positions contributing to the interface. This was achieved using cd-hit [42]. The residues used in clustering were defined according to Chothia *et al.* [28]: L34, L36, L38, L43, L44, L46, L87, L89, L98, L100, H35, H37, H39, H44, H45, H47, H91, H93, H103 and H105. Using a similarity threshold of 80%, we obtained 93 clusters, 37 of which contained less than three elements and were discarded to avoid the inclusion of immunoglobulins with unusual interfaces in our analysis. The immunoglobulins representing the centroid of each of the remaining 56 clusters were added to the 45 selected λ-type immunoglobulin structures to obtain the final dataset.

The structural similarity of the residues contributing to the interfaces and listed above was measured using lga software [43] in sequence-dependent mode with a 10 Å distance cut-off. The distances computed by lga were used to calculate the global distance test–high accuracy (GDT_HA) parameter: 

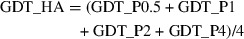

where GDT_P*n* denotes the percentage of residues that can be superimposed within a distance cut-off of *n* Å or less.

The GDT_HA values were employed to cluster the structures using the R package ‘cluster’ routine (M. Maechler *et al.*, unpublished results) with both diana (divisive) and hclust (agglomerative) methods. For agglomerative clustering, we used the ‘average’, ‘complete’, ‘ward’ and ‘single’ joining functions. For each clustering method, the optimal number of clusters was identified with the silhouette validation technique [31], which provides an estimate of the cluster tightness and separation, as implemented in the R package. The highest silhouette value (0.47) was obtained using the diana divisive clustering method with three clusters, one of which was formed by only one structure that was not included in the analysis (see Results section).

We used the automatic feature selection procedure already described in ref. [15] to select the sequence positions that have a significantly different residue distribution in antibodies belonging to different clusters, i.e. specific for a given type of interface. Each immunoglobulin was labeled according to the cluster it belonged to, and the Gini Impurity Index (as implemented in the Random Forest package [32,44]) was computed for each light and heavy chain residue. This index provides a relative ranking of the sequence positions on the basis of their ability to correctly discriminate the structural cluster to which an immunoglobulin belongs. The eight sequence positions with the highest Gini index are able to discriminate between the clusters with a classification error lower than 10%, and were manually analyzed.

In order to verify whether the difference in the packing geometry of immunoglobulins in the two clusters is reflected in a different geometry of their binding site, we measured the distances between the Cα of residues L55 and H57 and of residues L24 and H25 (which are the furthest structurally conserved residues in the antigen-binding site) and between the Cα of residue 36 of the light chain and of the last insertion before residue 101 of the heavy chain (this residue has a different Kabat–Chothia number according to the length of the H3 loop, and is called H100X here) for each immunoglobulin in our dataset. We used Pearson’s chi-squared test (as implemented in the R package) to verify whether they were statistically significantly different in immunoglobulins belonging to the two clusters.

We measured the volumes of the antigens bound to the immunoglobulin structures of our dataset, where present, using the Voronoi procedure, as implemented in the calc-volume program [45], with default parameters, and classified them into two groups according to whether their volume was smaller or larger than 505 Å^3^. This value corresponds to the first quartile of the antigen size distribution in our dataset. We calculated the *P* value for the hypothesis that immunoglobulins in a given cluster bind to smaller antigens by means of the hypergeometric cumulative distribution function, which measures the probability of finding at least as many antibodies binding to a small antigen in a cluster of similar size randomly extracted from the whole set of antibodies.

## Abbreviations

CDR: complementarity determining region
F(ab)2: two connected Fabs
Fab: antigen-binding fragment
Fv: variable fragment
GDT_HA: global distance test–high accuracy
PDB: Protein Data Bank
RMSD: root-mean-square deviation
VH: heavy chain variable domain
VL: light chain variable domain

## Supporting Information

Table S1 Antibody germline usage. Usage of IGLV/IGKV germline genes in immunoglobulins belonging to clusters A and B.

Please note: As a service to our authors and readers, this journalprovides supporting information supplied by the authors. Suchmaterials are peer-reviewed and may be re-organized for onlinedelivery, but are not copy-edited or typeset. Technical supportissues arising from supporting information (other than missingfiles) should be addressed to the authors.
