# Supplementary material for: The association of heavy and light chain variable domains in antibodies: implications for antigen specificity
Source: FEBS J. 2011 Aug;278(16):2858–66. doi: 10.1111/j.1742-4658.2011.08207.x (PMC3562479; doi:10.1111/j.1742-4658.2011.08207.x)
Supplement: Table S1 — Antibody germline usage. Usage of IGLV/IGKV germline genes in immunoglobulins belonging to clusters A and B. [file febs0278-2858-sd1.zip › ejb_8207_sm_febs_8207_sm_TableS1.pdf]

the FEBS Journal

www.febsjournal.org

The association of heavy and light chain variable domains in antibodies: implications for antigen specificity

Anna Chailyan, Paolo Marcatili and Anna Tramontano

DOI: 10.1111/j.1742-4658.2011.08207.x

Germline	Cluster A	Cluster B
**IGKV1-110**	4	0
**IGKV1-117**	3	0
**IGKV1-13**	1	0
**IGKV1-133**	2	0
**IGKV1-16**	1	0
**IGKV1-33**	1	0
**IGKV1-39**	1	0
**IGKV1-5**	2	0
**IGKV1-NL1**	1	0
**IGKV10-94**	0	2
**IGKV10-96**	0	4
**IGKV12-44**	2	0
**IGKV14-100**	0	1
**IGKV14-111**	1	0
**IGKV15-103**	1	0
**IGKV19-93**	1	0
**IGKV2-109**	1	0
**IGKV2-137**	2	0
**IGKV3-10**	1	0
**IGKV3-12**	2	0
**IGKV3-2**	1	0
**IGKV3-20**	3	0
**IGKV3-4**	1	0
**IGKV4-53**	1	0
**IGKV4-57**	2	0
**IGKV4-59**	3	0
**IGKV4-70**	1	0
**IGKV4-74**	1	0
**IGKV4-79**	1	0
**IGKV5-43**	1	0
**IGKV5-48**	1	0
**IGKV6-15**	1	0
**IGKV6-20**	1	0
**IGKV8-21**	2	0
**IGKV8-28**	1	0
**IGKV9-124**	0	1
**IGLV1**	0	23
**IGLV1-40**	3	0
**IGLV1-44**	2	0
**IGLV1-47**	2	0
**IGLV1-51**	3	0
**IGLV2-11**	2	0
**IGLV2-14**	1	0
**IGLV3**	3	0
**IGLV3-1**	1	0
**IGLV3-10**	1	0
**IGLV3-21**	1	0
**IGLV3-25**	1	0
**IGLV6-57**	1	0

Table S1 – Antibody germline usage. Usage of IGLV/IGKV germline genes in immunoglobulins belonging to clusters A and B.
